# Levofloxacin-Associated Neurotoxicity in a Patient with a High Concentration of Levofloxacin in the Blood and Cerebrospinal Fluid

**DOI:** 10.3390/antibiotics8020078

**Published:** 2019-06-12

**Authors:** Masashi Nishikubo, Maki Kanamori, Hiroaki Nishioka

**Affiliations:** Department of General Internal Medicine, Kobe City Medical Center General Hospital, Kobe, Hyogo 6500047, Japan; mnishi90@yahoo.co.jp (M.N.); maki1152@kcho.jp (M.K.)

**Keywords:** Levofloxacin, concentration, encephalopathy, myoclonus, hemodialysis

## Abstract

Neurotoxicity is a rare and intolerable adverse effect associated with levofloxacin therapy, whose diagnosis has mostly been reported based on medical history rather than quantitative measures in the blood. We report a 68-year-old man with levofloxacin-associated encephalopathy and myoclonus with high levels of levofloxacin in the blood and cerebrospinal fluid. After hemodialysis, these decreased, and his symptoms rapidly improved. An electroencephalogram was also normal. This case showed the concentration of levofloxacin to be clearly related to levofloxacin-associated neurotoxicity. Therefore, an estimation of its concentration may contribute to accurate diagnosis.

## 1. Introduction

Levofloxacin is a third-generation fluoroquinolone antibiotic used for treatment of a wide range of bacterial infections [1]. Neurotoxicity caused by levofloxacin is a known but rare adverse effect, with symptoms including headache, dizziness, sleep disturbance, psychosis, delirium, seizures, myoclonus, and others [2]. These symptoms are often intolerable and compel the discontinuation or change of treatment with levofloxacin. A high concentration of levofloxacin in the blood is believed to cause neurotoxicity, but few studies have specifically examined this in affected patients. Instead, nearly all cases have been diagnosed based on a history of prior exposure to levofloxacin, improvement after its cessation, and exclusion of other causes of neurotoxicity [3,4,5,6,7,8]. We report a case of levofloxacin-associated encephalopathy and myoclonus clearly related to high concentrations of levofloxacin in the blood and cerebrospinal fluid (CSF).

## 2. Case Presentation

A 68-year-old Japanese man was admitted to our hospital in winter due to disturbed consciousness and difficulty with body movements. Approximately two weeks prior, he had experienced fever and generalized edema. Thirteen days before admission, his family physician prescribed levofloxacin at 500 mg/day, furosemide at 20 mg/day, and spironolactone at 25 mg/day for seven days. The patient complied with this and became afebrile, and his edema disappeared. Two days before admission, his wife noticed that his speech was slurred, and he was only able to understand short and simple sentences. On the day of admission, he could not communicate and was unable to speak comprehensibly. He could not move by himself. His past medical history included cerebral palsy with lower limb atrophy, but he could walk using a frame. He had normal mental and intellectual development. He also had chronic kidney disease (CKD) at the stage of G4A3 due to nephrosclerosis and a horseshoe kidney, with a creatinine level of around 2.5 mg/dL. He had been treated with nifedipine, doxazosin, and febuxostat and did not use any over-the-counter supplements or illegal drugs. 

Upon physical examination, his blood pressure was 65/40 mm Hg, his heart rate was 40 bpm, his respiratory rate was 20 breaths/min, and his body temperature was 27.9 °C. His consciousness was registered as E4V1M4 (E: Eye opening, V: Best verbal response, M: Best motor response) on the Glasgow Coma Scale. Accidental hypothermia was suspected, because his room had been cold and he had stayed there throughout the night. He was rewarmed immediately, and his body temperature increased to 35.7 °C, after which his vital signs became stable. However, his consciousness level remained abnormal, and he also showed myoclonus in both upper limbs. The deep tendon reflex of the biceps was normal. Laboratory findings showed a white cell count of 2700/μL, a hemoglobin level of 5.9 g/dL, a platelet count of 16.5 × 10^4^/μL, albumin at 2.0 g/dL, aspartate aminotransferase at 137 U/L, alanine aminotransferase at 50 U/L, alkaline phosphatase at 288 U/L, γ-glutamyl transpeptidase at 13 U/L, blood urea nitrogen at 82.3 mg/dL, creatinine at 3.84 mg/dL, sodium at 142 mEq/L, calcium at 8.0 mg/dL, glucose at 101 mg/dL, thyroid-stimulating hormone at 9.29 μU/mL, free T4 at 1.03 ng/mL, and vitamin B1 at 3.9 g/dL. Antithyroid peroxidase antibody and antithyroglobulin antibody were undetectable. An arterial blood gas analysis did not show CO_2_ elevation and indicated mild metabolic acidosis. An electrocardiogram initially showed first-degree atrioventricular block, which became normal sinus rhythm after rewarming. Head-computed tomography and magnetic resonance imaging examinations showed no abnormalities, and an electroencephalogram showed generalized slowing waves. CSF was shown to have a cell count of 0/μL, a protein concentration of 68 mg/dL, and a glucose level of 65 mg/dL. Levofloxacin-associated encephalopathy and myoclonus were suspected based on the patient’s history of high levofloxacin dosages and present renal function. 

**Figure 1:**
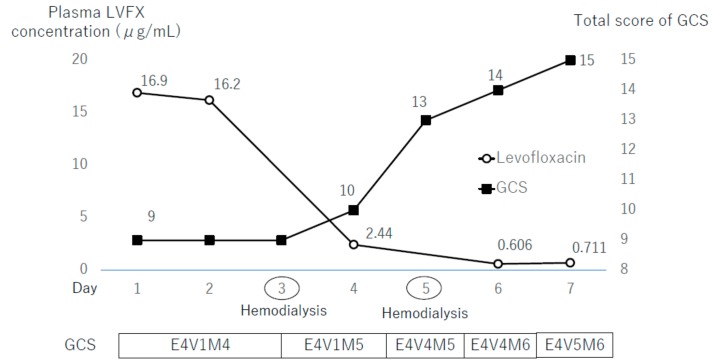
The patient’s clinical course. Hemodialysis was conducted three and five days after hospital admission. LVFX: Levofloxacin; GCS: Glasgow Coma Scale; E: Eye opening; V: Best verbal response; M: Best motor response.

The patient was treated through the administration of fluids and furosemide with the aim of levofloxacin being excreted in his urine. Although the patient produced more urine, his creatinine did not return to basal level, and his consciousness level did not improve. Hemodialysis was carried out on days 3 and 5 after admission, and his consciousness level improved and completely recovered by day 7 (Figure 1). The myoclonus disappeared four days after admission. An electroencephalogram on day 15 showed no slowing waves. His Naranjo adverse drug reaction probability scale registered at 7 points, which indicated a probable relationship between his symptoms and adverse drug reactions to levofloxacin [9]. Sixteen days after admission, the patient was transferred to another hospital for rehabilitation. Later, we measured the concentration of levofloxacin in the reserved samples of plasma or CSF, obtained during hospitalization. The plasma concentrations of levofloxacin were 16.9, 16.2, 2.44, 0.61, and 0.71 μg/mL on days 1, 2, 4, 6, and 7 after admission, respectively (Figure 1). The initial CSF concentration of levofloxacin was 6.81 μg/mL.

## 3. Discussion

The clinical course of this patient yielded two important observations. First, a high concentration of levofloxacin in the blood and CSF was observed in conjunction with levofloxacin-associated encephalopathy and myoclonus. Second, hemodialysis was useful in the rapid resolution of levofloxacin-associated neurotoxicity. 

Currently, a daily dose of 500 mg or 750 mg of levofloxacin is approved in many countries and is widely used in patients with normal renal function. In healthy volunteers receiving levofloxacin at single doses of 500 mg, 1000 mg, or 1500 mg, the mean maximum drug concentration (*Cmax*) values in plasma were 5.43 ± 1.76, 10.2 ± 2.92, and 14.6 ± 3.49 μg/mL ± SD, respectively [10]. In the same subjects, the mean time of *Cmax* (*Tmax*) values in plasma were 1.7 ± 0.5, 2.0 ± 0.9, and 2.2 ± 1.0 hours ± SD [10]. Another study in individuals with normal renal function reported that after daily oral administration of 500 mg of levofloxacin for seven days, the *Cmax* value was almost the same as that of the first day [11]. Scotton et al. examined the CSF concentration of levofloxacin in five patients with acute bacterial meningitis who were treated with β-lactam plus levofloxacin (500 mg twice a day. or once a day.) and found a concentration of 1.99 ± 0.67 μg/mL two hours after dosing without complications [12]. Levofloxacin is excreted from the kidneys via urine [13]. If patients have renal impairment, a dose reduction is urgently necessary in order to avoid the accumulation of levofloxacin and related adverse effects [13]. In fact, impaired renal function has been reported to be a predisposing factor for levofloxacin-associated neurotoxicity [4,5,6,7,8]. However, reports on the concentration of levofloxacin in blood or CSF in patients with levofloxacin-associated neurotoxicity are rare. Our patient had impaired renal function and higher levofloxacin concentrations in both his blood and CSF, even six days after cessation of levofloxacin treatment. This clearly indicated its association with his development of neurotoxicity. Although the safe-limit concentration of levofloxacin in blood has not been established, based on previous reports as well as our current case, we hypothesized that over 15 μg/mL of levofloxacin for some days in the blood might be related to the development of levofloxacin-associated neurotoxicity. The neurotoxic potential of levofloxacin is closely related to its activity on gamma-aminobutyric acid (GABA) receptors and *N*-Methyl-D-aspartate (NMDA) receptors. Levofloxacin inhibits GABA-A receptors and activates NMDA receptors [14]. A high level of levofloxacin may cause excessive neuronal excitatory signaling, leading to neurotoxic side effects such as encephalopathy and myoclonus. [15] 

Normally, treatment for drug-associated neurotoxicity involves the cessation of the causative agents. In previous reports on quinolone-associated encephalopathy, including levofloxacin, rapid improvement was achieved by discontinuation of quinolones only [3,4,5,6,7,8], and hemodialysis is not routinely recommended [15]. However, this was not the case for the patient in this study, for whom dramatic improvement in his consciousness level and myoclonus was achieved only after hemodialysis. The fact that discontinuation alone did not yield improvement may have been due to delayed renal clearance as a result of acute kidney injury and CKD. After hemodialysis, the plasma concentration of levofloxacin decreased, the patient’s consciousness level quickly improved, and myoclonus disappeared. This approach is supported by two additional reported cases of levofloxacin-associated neurotoxicity that showed rapid resolution after hemodialysis in patients with impaired renal function [16].

## 4. Conclusions

A high concentration of levofloxacin in the blood and CSF may be related to levofloxacin-associated encephalopathy and myoclonus and should be included in an examination for accurate diagnosis. Hemodialysis may be a useful treatment for levofloxacin-associated encephalopathy in patients with impaired renal function.
